# Experimental and Computational Analysis of High-Intensity Focused Ultrasound Thermal Ablation in Breast Cancer Cells: Monolayers vs. Spheroids

**DOI:** 10.3390/cancers16071274

**Published:** 2024-03-25

**Authors:** Heba M. Badawe, Jean Paul Harouz, Petra Raad, Kareem Abu, Anthony Freije, Kamel Ghali, Wassim Abou-Kheir, Massoud L. Khraiche

**Affiliations:** 1 Neural Engineering and Nanobiosensors Group, Biomedical Engineering Program, Maroun Semaan Faculty of Engineering and Architecture, American University of Beirut, Beirut 1107 2020, Lebanon; hmb28@mail.aub.edu (H.M.B.); kja15@mail.aub.edu (K.A.); asf17@aub.edu.lb (A.F.); 2 Department of Mechanical Engineering, Maroun Semaan Faculty of Engineering and Architecture, American University of Beirut, Beirut 1107 2020, Lebanon; jeh15@mail.aub.edu (J.P.H.); ka04@aub.edu.lb (K.G.); 3 Department of Anatomy, Cell Biology and Physiological Sciences, Faculty of Medicine, American University of Beirut, Beirut 1107 2020, Lebanon; wa12@aub.edu.lb

**Keywords:** HIFU, thermal ablation, 2D monolayer, 3D spheroids, temperature increase

## Abstract

**Simple Summary:**

Breast cancer is a significant global health challenge, ranking as the second leading cause of death worldwide. Current treatment modalities, such as invasive surgery and chemotherapy, while effective to some extent, pose notable risks to patient well-being. High-intensity focused ultrasound (HIFU) offers a non-invasive alternative, utilizing precise acoustic energy for targeted tumor ablation while sparing surrounding healthy tissues. However, HIFU’s efficacy depends on various factors, necessitating comprehensive research for optimization. The research focuses on in vitro thermal ablation of epithelial breast cancer cell lines, exploring the impact of HIFU on both 2D monolayer and 3D spheroidal cell configurations. The investigation assesses ultrasound parameters, including acoustic intensity, duty cycle, and sonication duration, evaluating their influence on temperature elevation and tumor cell ablation. Empirical findings are compared with numerical simulations, contributing to a deeper understanding of HIFU’s potential in breast cancer treatment and paving the way for more effective therapeutic strategies.

**Abstract:**

High-intensity focused ultrasound (HIFU) is a non-invasive therapeutic modality that uses precise acoustic energy to ablate cancerous tissues through coagulative necrosis. In this context, we investigate the efficacy of HIFU ablation in two distinct cellular configurations, namely 2D monolayers and 3D spheroids of epithelial breast cancer cell lines (MDA-MB 231 and MCF7). The primary objective is to compare the response of these two in vitro models to HIFU while measuring their ablation percentages and temperature elevation levels. HIFU was systematically applied to the cell cultures, varying ultrasound intensity and duty cycle during different sonication sessions. The results indicate that the degree of ablation is highly influenced by the duty cycle, with higher duty cycles resulting in greater ablation percentages, while sonication duration has a minimal impact. Numerical simulations validate experimental observations, highlighting a significant disparity in the response of 2D monolayers and 3D spheroids to HIFU treatment. Specifically, tumor spheroids require lower temperature elevations for effective ablation, and their ablation percentage significantly increases with elevated duty cycles. This study contributes to a comprehensive understanding of acoustic energy conversion within the biological system during HIFU treatment for 2D versus 3D ablation targets, holding potential implications for refining and personalizing breast cancer therapeutic strategies.

## 1. Introduction

Cancer poses a significant global public health challenge and currently ranks as the second leading cause of death worldwide, following cardiovascular diseases. Notably, among women, breast cancer emerges as the most extensively studied malignancy and a foremost contributor to cancer-related fatalities [1]. In general, breast tissues are categorized as either dense or fatty, depending on the ratio of the density of glandular and connective tissue to adipose tissue, which comprises the mammographic density (MD) [2]. Cancerous breast tumors exhibit variations in size, volume, and density, corresponding to a high MD, necessitating adequate demarcation of their boundaries to ensure precise target volumes for targeted therapies [3,4].

Conventional cancer treatments, such as surgeries, carry significant risks, including infection, bleeding, and scarring, given their level of invasiveness. Add to that, chemotherapy and immunotherapy can cause systemic side effects such as nausea, hair loss, and immune-related adverse events while requiring extended recovery periods [5]. Other less traditional yet minimally invasive thermal ablation techniques include laser ablation, microwave ablation, radio frequency ablation, and high-intensity focused ultrasound (HIFU) ablation [6]. Each of these techniques focuses an energy source at a designated target volume of malignant cells to generate intense heat, causing coagulative necrosis and leading to irreversible cell damage. Such treatment methods are mostly effective in early-stage breast cancer cases where the diameter of a tumor does not exceed 2 cm [7,8]. Nevertheless, challenges in the effectiveness of such thermal ablation techniques persist, including the precise delineation of the tumor based on its size, depth, and type of breast tissue to sparse the surrounding, viable healthy cells in the fringe heating region [9,10], tumor regrowth at the rim of the ablation zone, and limitations in addressing larger malignant volumes [11].

Ultrasound is a non-invasive modality widely utilized in biomedical engineering for a diverse range of applications, which include imaging, neuromodulation [12], and ablation [13]. Specifically, high-intensity focused ultrasound (HIFU) has emerged as a promising alternative in the realm of non-invasive and non-ionizing ablation methods. This innovative approach facilitates the precise ablation of deep tumor cells without causing harm to the underlying skin or adjacent structures along the path of the acoustic beams. HIFU achieves this by inducing coagulative necrosis within the body, showcasing its potential as a safe and effective therapeutic tool in medical practice [14]. In general, HIFU induces two major biological effects on cell ablation: thermal and non-thermal [15]. Thermal ablation results from the conversion of acoustic energy to thermal energy at the target site through an increase in the energy density, thus elevating the temperature of the cells to a range of 60–85 °C within seconds [16]. These elevated temperatures within the focal zone initiate protein coagulation and cell membrane fusion, ultimately leading to the necrosis of tumor cells. Beyond this focal region, at the tumor–healthy cell interface, heat diffusion generates a temperature gradient, where cells do not experience an instantaneous fatal thermal dose but are subjected to temperatures exceeding 40 °C, where the regeneration of healthy cells initiates an immune response to sustain ongoing heating, aiming to inhibit, prevent, and confine further accumulation of thermal damage within the specified ablation boundaries [17]. In contrast, mechanical or non-thermal ablation involves the transfer of acoustic power by HIFU waves, inducing cavitation at the target site and resulting in the mechanical disruption of cell membranes. High-pressure acoustic waves induce changes in the gaseous composition within tissues, instigating oscillation and subsequent rupture of bubbles, causing subcellular-scale mechanical damage to tissues [18].

HIFU has been successfully employed in the treatment of patients who have cancerous tumors in the liver, breast, kidney, and pancreas [19]. Yet, this technology still requires adequate research and study. Its efficacy in tumor ablation relies on a multitude of factors, contingent upon the ultrasound parameters employed and the specific target areas, all of which significantly influence its success rate. Consequently, there is a pressing imperative for comprehensive exploration and refinement of HIFU technology to optimize therapeutic outcomes. Enhancing the efficiency of HIFU tumor treatment necessitates thorough in vitro assessments involving the cultivation of tumor cells for preclinical cancer research. Traditional cell culturing methods involve the growth of cells in monolayers, which replicate along a single plane. The advantages of this 2D cell culture model include its simplicity, cost-effectiveness, and ease of maintenance, making it suitable for basic cancer studies. Nevertheless, this two-dimensional cell model fails to replicate the intricate cell–cell and cell–extracellular environment interactions crucial for a comprehensive understanding of natural cell behavior in vivo. These interactions encompass processes such as differentiation, proliferation, gene and protein expression, and cellular responses to external stimuli [20]. To overcome the limitations inherent in the two-dimensional model, researchers turn to spheroid models. Spheroids offer a three-dimensional configuration that permits cells to interact and proliferate in all directions, fostering the development of an extracellular architecture with a layered structure and a proliferative profile [21]. This 3D environment accounts for the presence of oxygen and nutrients, allowing cells to grow with a gene expression profile closely resembling that of tumor cells. Consequently, the use of spheroid 3D cell configurations offers a more accurate evaluation of biological responses compared to the conventional 2D monolayer cell formation [22]. Nevertheless, both cell configurations serve as potential in vitro models for evaluating ultrasound-based therapies and can be employed to validate various therapeutic applications.

In this study, our aim is to investigate thermal cellular ablation using a more representative in vitro model, specifically 3D spheroids, and contrast it with the commonly utilized 2D monolayers model. To that end, we employed both experimental and computational methodologies to investigate how the two cell cultures would interact with acoustic waves under the impact of ultrasound parameters, specifically acoustic intensity, duty cycle (DC), and sonication duration (SD), on the extent of temperature elevation and tumor cell ablation. Furthermore, we compared and validated our empirical findings with the results from numerical simulations using Pennes’ bioheat computational model. Our study focused on the in vitro thermal ablation of epithelial breast cancer cell lines using both experimental and computational approaches, which may offer novel insights into optimizing HIFU treatment parameters and understanding cellular responses.

## 2. Materials and Methods

### 2.1. HIFU Mapping and Characterization

**Figure 1:**
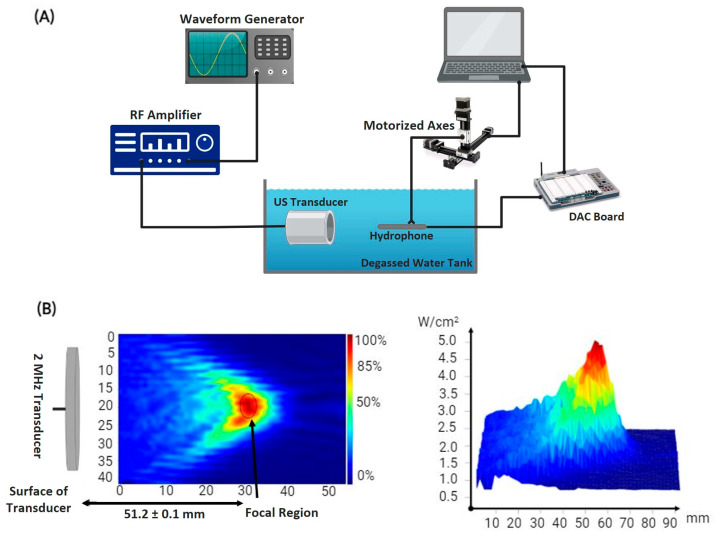
Acoustic profile setup. (**A**) Schematic diagram of the experimental setup used to determine the acoustic intensity of HIFU emitted by the transducer and the temperature rise at the target. (**B**) Heat map displaying the intensity distribution at the focal point of the transducer present 51.2 mm away from the center of curvature of its surface.

Characterizing the ultrasound transducer required determining its power output per unit volume and focal point. The utilized transducer of a 2 MHz center frequency (SU101-019; Sonic Concepts, Inc., Bothell, WA, USA) generated HIFU waves after receiving a sinusoidal electrical signal from a function generator (SIGILENT-SDS 1025), pre-amplified by a 50-ohm RF power amplifier (100 L Broadband Power Amplifier, Electronics & Innovation Ltd., Rochester, NY, USA). Generally, the transducer converted the electrical signals into propagating mechanical ultrasonic waves that were captured by an immersible needle hydrophone (Onda HNR-0500) of size 37.3 mm × 2.5 mm. The latter converted the signals back into voltage recordings to be stored in a data acquisition system, followed by processing and quantization into acoustic intensity [23]. The hydrophone was originally mounted on a 3D-motorized axes system, allowing for systematic movement into different positions relative to the transducer to scan a definite geometric volume (Figure 1A). Using this transmission method, the focal point of the ultrasound transducer, where the maximum spatial peak temporal average intensity of 5 $W/{cm}^{2}$ was recorded, was localized at 51.2 ± 0.1 mm from the face of the transducer in the free-field tank filled with degassed distilled water (Figure 1B).

### 2.2. Temperature Acquisition

**Figure 2:**
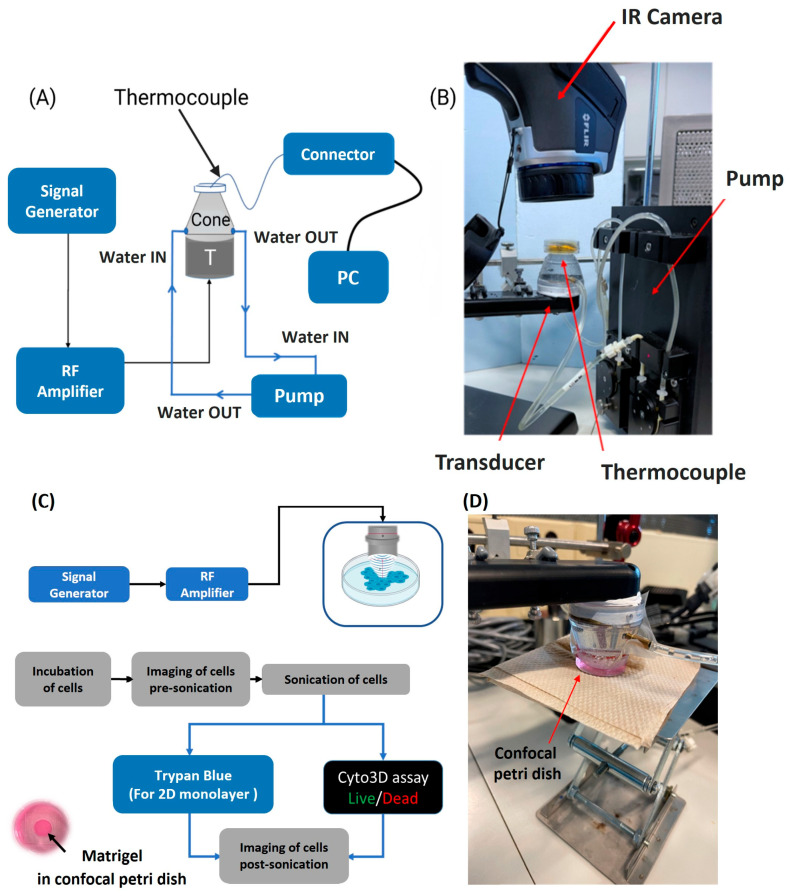
Temperature measurements and ultrasound ablation setup. (**A**) Schematic diagram of the setup to measure the temperature. The thermocouple was taped at the bottom of the confocal Petri dish to record the maximum temperature reached at the end of sonication. (**B**) Photo of the experimental setup, the temperature at and around the focus was captured by the IR camera. (**C**) Schematic diagram of the experimental setup where cultured tumor cells are treated with HIFU at distinct sets of ultrasound parameters and then stained and visualized under the microscope. (**D**) Photo of the ablation experimental setup in which cultured cells are sonicated with HIFU.

For temperature measurements in the focal region of the ultrasound transducer used in the 2D and 3D cell ablation experiments, a thin K-type thermocouple (40 AWG/0.08 mm; Omega, Newark, NJ, USA) of 0.08 mm thickness was taped in place, using Poly-IMID tape, at the bottom of a confocal Petri dish, positioned within the focal region of the ultrasound transducer (Figure 2A). The 0.08 mm diameter of the thermocouple is small relative to the 5 mm depth of the focal area of the 2 MHz ultrasound transducer. Therefore, the thermocouple constituted negligible obstruction in the path of ultrasound propagation. Indeed, the motion of the thermocouple at the ultrasound focal area can cause viscous heating artifacts, rapidly increasing the temperature readings [24,25]. To ensure artifact delay, we used short ultrasound pulses of less than 100 ms, beginning temperature acquisition 500 ms after the start of sonication, as indicated by [26].

The confocal Petri dish had a 35 mm glass bottom slide with a 10 mm micro-well, suitable for long-term cell culture, and could withstand very high temperatures. A fine-wire type K-thermocouple, with a standard accuracy ranging from ±1.5 °C to ±2.5 °C, was used to decrease temperature measurement errors that could arise from viscous heating and thermal conductivity artifacts [27,28]. To further ensure accurate temperature measurement, the Petri dish/cell culture was placed on an insulating material made from cellulose. Cellulose materials typically have low thermal conductivity in the order of $10^{-2}\;W/m\cdot K$ [29]. The Petri dish was not in contact with any external factors during sonication to prevent any potential thermal exchange between the Petri dish and its immediate environment.

For the delivery of ultrasound waves, a coupling cone, sealed with an acoustically transparent membrane, was placed over the inverted ultrasound probe so that the acoustic focal point was within 1 mm from the tip of the probe, knowing that the focal length of the ultrasound transducer is 5 mm. To cool the transducer off and remove any potential gas bubbles that may form within the cone, deionized, degassed water was continuously pumped in and out of the cone using a pump perfusion kit (PPS2, Multichannel systems) at a maximum flow rate of 30 mL/min. The cone was then coupled to the confocal Petri dish containing the glass micro-well. An IR-FLIR E40 Thermal Imaging Camera with a 0.07 °C thermal sensitivity was also vertically mounted over the Petri dish (Figure 2B) to collect quantitative data for the examination of any temperature variation within and outside the focal region of the focused ultrasonic transducer.

### 2.3. Two-Dimensional and Three-Dimensional Tumor Cell Culturing

MDA-MB 231 and MCF7 epithelial breast cancer cell lines, obtained from the American Type Culture Collection (ATCC, Manassas, VA, USA), were considered for this study. The cells were cultured in a glucose-rich DMEM medium containing 10% fetal bovine serum (FBS) (Sigma, St. Louis, MO, USA, F-9665) and 1% penicillin/streptomycin (Lonza, Basel, Switzerland, DE16–602E) with 5% carbon dioxide in a humidified incubator at a temperature of 37 °C [30]. The medium was replenished after 36 h to remove any waste products. Around 3000 cells were plated in the glass micro-wells of the confocal Petri dish and incubated for 24–36 h before ultrasound ablation. The cells were ready for treatment after reaching a confluence of more than 95%. Throughout the process, from seeding to treatment, the cells were checked under an inverted bright field microscope to ensure healthy proliferation and the absence of contamination.

The characteristics of sphere-like MCF-7 and MDA-MB 231 breast cancer cells were studied using a sphere formation assay. In this assay, 3D tumor spheroids that mimic the environment of the extracellular matrix (ECM) were formed. The single cells were mixed with a 1:1 combination of cold Matrigel (a growth factor-reduced substance, including necessary proteins and cytokines) with serum-free StableCell TM RPMI-1640 at a density of 5000 cells/dish. The mixture was poured as droplets in micro-wells present in the center of the small Petri dishes and allowed to solidify at 37 °C in a humidified incubator with 5% CO_2_ for 1 h. Afterward, 1.5 mL of StableCell TM RPMI-1640 cell growth medium containing 5% FBS was added to each Petri dish. The medium was replaced every 2–3 days until the spheroids matured and were ready for further experimentation, specifically ultrasound sonication. The cells were incubated for a total of 9 days while maintaining a consistent incubation period across all experiments to ensure consistency.

### 2.4. Ultrasound Sonication and Cellular Ablation

Before any treatment, the cells were examined in each Petri dish to ensure even growth, and pictures were taken using a bright field microscope. To measure the ablation area percentage, a glass slide with nine small squares, each measuring 100 × 100 ${µm}^{2}$, was placed under the cells. These squares were positioned so that one covered the middle of the focal region while the other eight covered spots scattered around and outside the treatment zone. ImageJ software was used to count the cells. Ultrasound sonication of the cultured cells was carried out using an ultrasound transducer coupled with a cone filled with degassed water and aligned with the middle of the glass micro-wells of the confocal Petri dish containing the cells along with their culture media (Figure 2C,D). To ensure optimal transmission of ultrasound waves, a layer of Phosphate-Buffered Saline (PBS) solution was added on top in cases where the culture media was insufficient. The ultrasound transducer had a focal length of 5 mm, and the focal point was positioned 1 mm from the tip of the coupling cone. Utilizing an acoustically transparent Petri dish helped minimize the generation of standing waves when sonicating the cells with HIFU [31]. Acoustically transparent materials allow ultrasound waves to pass through without significant reflection or interference, reducing the likelihood of standing wave formation while ensuring a more uniform and controlled application of ultrasound energy to the cells during sonication, improving the precision and effectiveness of the procedure. Add to that, the wavelength of the ultrasound wave at 2 MHz was small compared to the dimensions of the micro-wells, decreasing the likelihood of standing wave formation [32]. Bright field and fluorescent images of cellular ablation were taken at different magnifications post ultrasound sonication.

Post ultrasound sonication, cellular viability was determined using two staining techniques: Trypan Blue Exclusion and the Fluorescent Cyto3D Live/Dead Assay. Trypan Blue, a straightforward and user-friendly stain, was utilized. Upon application, it rendered living cells unstained, while nonviable cells appeared distinctly blue when observed under a microscope. The Fluorescent Cyto3D Live/Dead Assay is a non-toxic nucleic dye that marks damaged cancer cells in red using Acridine Orange (AO), and alive cancer cells in green with Propidium Iodide [33]. This dye was applied before and after treating the tumor cells with ultrasound sonication. For both the 2D and 3D cell cultures, the dye had to be mixed with pre-prepared media containing 2 µL of Cyto3D for every 100 µL of media. This mixture was prepared in a sterile, light-sensitive environment to prevent any signal loss. After completely removing the media from the Petri dish, 600 µL of the prepared solution was added directly to the Matrigel and incubated for at least an hour to allow the chemicals to permeate through the gel and into the cell colonies and spheres.

For cell visualization, an inverted microscope was employed to capture bright field and/or fluorescent images. Cells were also examined for viability pre-treatment to guarantee that cell death was solely due to the ultrasound treatment. Cell quantification was conducted on 3D Matrigel cultures using the following procedure: Initially, all the culture medium was completely withdrawn and transferred to a conical tube, which already contained previously collected media from the Petri dish. Next, cold trypsin was introduced to the Petri dish and allowed to act for approximately 5 min. Following that, the cells were gathered and displaced from the dish to the same conical tube. After thoroughly mixing the contents, 50 µL was drawn from the conical tube and combined with 50 µL of Trypan Blue. Finally, 10 µL of the resultant mixture was loaded onto the hemocytometer for the purpose of cell counting.

### 2.5. Statistical Analysis

Ablation data were obtained across four spatial peak pulse average intensities covering $146.7\;W/{cm}^{2}$ to $500\;W/{cm}^{2}$ for each DC spanning from 15% to 55%. The ablation data were analyzed with non-parametric tests using IBM SPSS Statistics (29th version), given the small sample size per DC and where the data were not normally distributed. Multiple pairwise comparisons were conducted utilizing the Kruskal–Wallis test and Dune-Bonferroni corrected to determine significant differences in cell ablation among different DCs for a specified SD. Additionally, within a particular DC, two independent ablation samples from different SDs were compared using the Mann–Whitney U-test. Combining all duty cycles, the Mann–Whitney U-test was used to assess differences in ablation levels at the focal area caused by SDs of 5 min and 10 min. Statistical significance was determined at *p* < 0.05.

### 2.6. Computational Modeling

We developed mathematical models to assess the thermal response of both monolayer and spheroid cell cultures to pulsed HIFU acoustic sonication for results validation and outcome predictions. The models were validated with experimental data and used to conduct a parametric study for the different operating conditions, such as DC and maximum pressure, to determine the effect of HIFU on cellular ablation. The wave propagation model provided the acoustic field distribution and the corresponding thermal energy deposition in the cells. The thermal energy was used in the thermal model to determine the temperature distribution in the cell cultures and their corresponding ablation rate.

#### 2.6.1. Computational Domain

**Figure 3:**
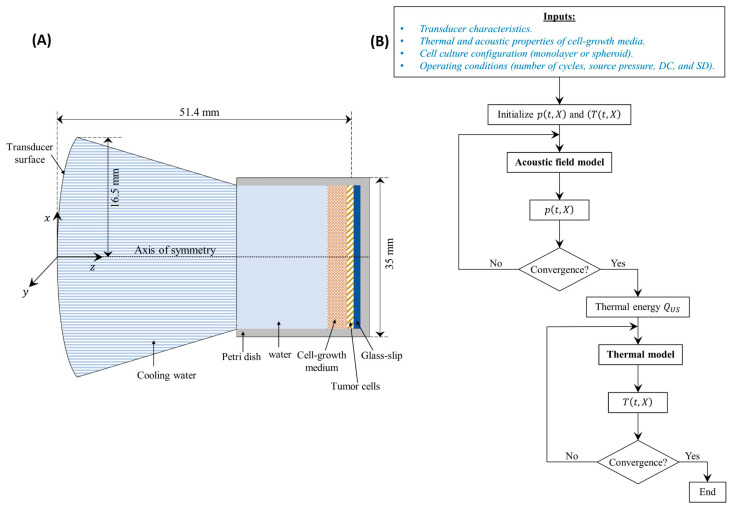
Computational model. (**A**) Schematic diagram of the computational domain adopted for the numerical simulations. (**B**) Adopted solution flowchart for the developed mathematical models.

The developed model was used to determine the temperature distribution inside the cell cultures of both configurations, monolayer and spheroids. For this reason, the adopted computational domain, presented in Figure 3A, consisted of a Petri dish and a transducer surface. The Petri dish in each configuration was formed of different layers, starting from the transducer surface, as follows: cooling water, cell growth medium, cells, glass, and the Petri dish bottom surface. The model did not take into account any mixing between the layers. To simplify the computational domain for the spheroids cell culture, it was assumed that they occupy a homogenous layer, neglecting the voids created between the different hydrogel spheres where the cells were seeded. This could be adopted due to the relatively small (~μm) dimensions of those spheres. Moreover, the cooling water was separated from the cell culture by a thin polymeric membrane that was disregarded in the computations since it is basically acoustically transparent [34,35].

#### 2.6.2. Acoustic Field Model Coupled with the Thermal Model

Ultrasound waves emitted from the transducer traveled through the cooling water before passing through the culture medium where the cells were grown. At the interface of each layer, part of the acoustic energy was absorbed while the remaining energy was reflected. The fraction of reflected energy depended on the difference in the various layers of acoustic impedance (Z ($Kg/m^{2}\cdot s$)), which is the product of the layer’s density (ρ ($Kg/m^{3}$)) and acoustic wave velocity ($c_{0}(m/s)$) [36]. Within a single layer, the pressure fluctuations caused shearing of the cells, resulting in increased mechanical friction that was converted to thermal energy [37,38].

To model the wave propagation inside the cell cultures, the acoustic pressure field p(t,X)(Pa) was determined [39]. The Westervelt equation was adopted as it provided a more general acoustic field model to the commonly used Khokhlov–Zabozotskaya–Kuznetsov (KZK) equation [40]. It has also been used to model problems with standing wave conditions [41,42]. The Westervelt equation is derived from the fluid motion equations, making it more accurate than the KZK equation, which suffers from a validity region [43]. Such a model has been commonly adopted in medical ultrasound as it combines the influence of absorption, non-linearity, and diffraction mechanisms that are present in biological media [44] and given as follows:

(1) $$\frac{\delta }{c_{0}^{4}}\frac{{𝜕}^{3}p}{𝜕t^{3}}+\frac{\beta }{\rho c_{0}^{4}}\frac{{𝜕}^{2}p^{2}}{{𝜕}^{2}t^{2}}-\left(\frac{1}{c_{0}^{2}}\frac{{𝜕}^{2}p}{𝜕t^{2}}-\nabla^{2}p\right)=0,$$

The first term of Equation (1) describes the loss caused by the viscosity and heat conduction in the fluid. The second term describes the non-linear distortion of the propagation wave, and the last two terms represent the linear lossless wave propagation. The different terms of the equation represent the following: $\nabla^{2}$ ($m^{-2}$) is the Laplace operator, and $\beta (=1+\frac{B}{2A})$ is the coefficient of non-linearity, which is a function of the non-linearity parameter ($\frac{B}{A}$) of the medium. Acoustic diffusivity is represented by $\delta (=\frac{2{c_{0}}^{3}\alpha }{\omega })\;(m^{2}/s)$, which depends on the angular frequency (ω (rad/s)) of the source and the absorption/attenuation coefficient (α (Np/m)). The latter is determined by the frequency-dependent power law given as follows [45]:

(2) $$\alpha =\alpha_{ref}{\left(\frac{f}{f_{ref}}\right)}^{\eta },$$

where ${\;\alpha }_{ref\;}(Np/m)$ is the attenuation coefficient at a reference frequency ${\;f}_{ref\;}$ of 1 MHz and η is the attenuation exponent of the power law, and it depends on the type of medium [46].

Once the acoustic pressure p(t,X) was determined at each point X(x,y,z) of the cell culture domain, the resulting heat (thermal energy) deposition from the ultrasound wave $\left(Q_{US}\;(W/m^{3})\right)$ was computed from the acoustic intensity $\left(I\;(W/m^{2})\right)$ and the medium impedance (Z) using Equation (3):

(3) $$Q_{US}=2\alpha I=\alpha \frac{p^{2}\left(t,X\right)}{Z},$$

The model entailed an initial condition of acoustic pressure, which was set to zero at the beginning of sonication. Additionally, a set of boundary conditions was required to solve for the acoustic field. The geometry of the Petri dish enabled an axisymmetric solution around the z-axis, reducing the computational domain. Hence, a symmetry boundary condition was adopted at planes x=y=0. The transducer emitted a pulsed HIFU beam at z=0 [47], which was expressed as follows:

(4) $$\begin{array}{c} \left\{\begin{array}{c} \frac{p_{0}}{\sqrt{1+\frac{r^{2}}{d^{2}}}}\sin\left(t+\frac{d}{c_{0}}\left(\sqrt{1+\frac{r^{2}}{d^{2}}}-1\right)\right)\\ 0\;\;\;\;\;\;\;\;\;\;\;\;\;\;\;\;\;\;\;\;\;\;\;\;\;\;\;\;\;\; \end{array}\right. \end{array}\;\begin{array}{c} for\left\{\begin{array}{c} t\in \left[\left(n_{BP}-1\right)BP,\left(n_{BP}-1\right)BP+TBD\right]\;\\ r=\sqrt{x^{2}+y^{2}}\leq d*\tan\gamma \end{array}\right.\\ otherwise \end{array}$$

where $p_{0\;}(Pa$) is the pressure at the transducer surface,$\;n_{BP\;}$ is the number of burst cycles with a period of TBD (s) each during the total sonication duration.  r (m) is the radial distance from the center of the transducer with a focal distance d (m), within the plane $z=0.\;\gamma =\frac{\alpha }{\sqrt{1+\frac{r^{2}}{d^{2}}}}\;$, with *γ* (rad), is the aperture angle which is a function of the transducer radius  a (m)  and the focal distance d (m).

Finally, since the computational domain was finite, the perfectly matched layer boundary condition was adopted at the bottom of the Petri dish (end surface) to control the acoustic reflections from the end of the domain using artificial absorption [48].

Once the acoustic pressure was established in the Petri dish, the temperature distribution could then be determined from the thermal model. The energy balance presented in the bioheat model of Pennes’ was commonly used in these applications [49]. Pennes’ bioheat model is known for its simplicity, making it computationally efficient while capturing the essential mechanisms of heat transfer in biological tissues. This characteristic is particularly advantageous when dealing with complex simulations, allowing for quicker and more practical computations. Pennes’ model incorporates key physiological parameters, such as blood perfusion and metabolic heat generation ($Q_{m}$(W/$m^{3}$)), along with Fourier law for heat diffusion, which are crucial in modeling heat transfer in living tissues. The model assumes homogeneous and anisotropic biological tissue with constant blood perfusion with uniform blood temperature and, most importantly, constant thermal properties. More detailed bioheat models have been proposed in the literature to account primarily for the blood perfusion models that do not assume constant blood flow rates throughout the cells. This is especially important as tumors frequently display irregular and disordered development of blood vessels, resulting in diverse patterns of blood perfusion [50]. This uneven perfusion has significant consequences for the tumor microenvironment, impacting the delivery of oxygen and nutrients, removal of waste, and the overall temperature of the tissue [51]. Consequently, accurate modeling of blood perfusion is critical in assessing the effectiveness of thermal ablation techniques such as HIFU and microwave ablation [52]. Nonetheless, for the current model, the cells are not traversed by blood flow as they are treated in a Petri dish, whether in the monolayer or the spheroid configuration. Accordingly, the blood flow term will be neglected in the bioheat model, making thus the traditional Pennes’ bioheat model a suitable tool for the thermal distribution assessment in the cell cultures.

Moreover, as biological tissues are formed mainly from liquid water, their properties can be assumed to be constant within the limit of the liquid phase, which theoretically takes place at 100 °C at atmospheric pressure. In addition, Trujillo and Berjano showed that including the temperature dependence of the tissue thermal properties did not affect the thermal ablation results, with less than 12% error for the impedance change and less than 3.5% error in the lesion size prediction [53]. Accordingly, for the current study, the thermal properties of the tissues were considered independent of the temperature, as was adopted by many others. The temperature distribution ((T(t,X) (K)) was thus computed from Equation (5):

(5) $$\rho C\frac{𝜕T}{𝜕t}=k\nabla^{2}T-w_{b}C_{b}\left(T-T_{b}\right)+Q_{US}+Q_{m}$$

where C (J/kg·K)  and k (W/m·K) are the respective specific heat capacity and thermal conductivity of the medium. $C_{b}\left(J/Kg\cdot K\right),\;w_{b}\left(Kg/s\cdot m^{3}\right),$ and $T_{b}\;(K)$ are the specific heat capacity, perfusion rate and temperature of the blood, respectively. Among the different thermal energy sources in the bioheat equation, only the acoustic thermal energy deposition was considered. In fact, the absence of blood vessels in the cell culture eliminated the blood perfusion term, whereas the metabolic energy of the cells could be neglected due to its considerably low levels compared to ultrasound heating.

Similar to the acoustic field model, to solve the thermal model, a set of initial and boundary conditions were adopted. The cell culture, as well as the Petri dish, were considered initially at a uniform temperature equal to that of the laboratory (T(0,X)=25 °C). As for the boundary conditions, symmetry could be considered due to the nature of the problem with respect to the axis of the Petri dish that was aligned with the axis of the HIFU transducer. Therefore, a symmetry boundary condition was adopted at planes x=y=0. Moreover, a constant temperature was considered at the boundary of the dish, which was equal to that of the laboratory, given it was assumed that enough heat was dissipated to the ventilated lab to maintain a constant wall temperature.

Finally, thermal conduction between the cell culture and the adjacent glass slip and Petri dish as well as the heat diffusion in these layers was considered. To determine the temperature distribution in these layers, the three-dimensional transient-diffusion energy balance was considered as described by Incropera et al. [54] and was not reported here for conciseness.

The mathematical models of the wave propagation and temperature distribution were solved using the finite volume method with implicit backward Euler temporal scheme and central difference scheme for second-order spatial differentials. The non-linear and absorption terms of Equation (1) were solved as presented by Doinikov et al. [43] for a transient 3D Cartesian grid. To ensure the accuracy of the results with minimal computational time, the time step was $∆t=0.01/f_{0}\;$ with a grid of ∆x=∆y=0.2λ and ∆z=0.1λ [55], where $\lambda \;(=c_{0}/f_{0})\;(m)$ is the wavelength of the driving frequency of the transducer. Once a uniform pressure field was obtained at a steady-state (no change in the acoustic pressure with time), the acoustic intensity and the corresponding thermal energy generated in the cell cultures were determined and used in the thermal model with a time step of ∆t=0.01 s. Different time steps were adopted for the thermal model compared to the acoustic field model to reduce the computational time. They were considered similar to the findings obtained from the grid size and time step independence test conducted by Gupta et al. [39]. The convergence of the different parameters (p(t,X), T(t,X)) was reached when their calculated residuals between two consecutive iterations were less than $10^{-8}$. The different thermal and acoustic properties of the monolayer and spheroid cell cultures, as well as the different layers through which the acoustic wave was propagating, are presented in Table 1. Note that the missing acoustic parameters for either the cells or their growth medium were adopted as they were similar to those of water, which is the case for most biological tissues [36].

The solution of the developed mathematical model followed the flowchart presented in Figure 3B. The model inputs included the transducer characteristics $(a,\;d,f_{0},\;p_{0}),$ the cell cultures’ thermal and acoustic properties and configuration (monolayer and spheroid) and the operating conditions (TBD, DC, and SD). It started by initializing the pressure and temperature fields and used the inputs to determine the converged steady-state acoustic pressure distribution from the acoustic field model. The latter was incorporated to determine the thermal energy deposition ($Q_{US}$) that was inputted in the thermal model to conclude the converged temperature distribution.

## 3. Results

### 3.1. Focal Area and Temperature Measurements

**Figure 4:**
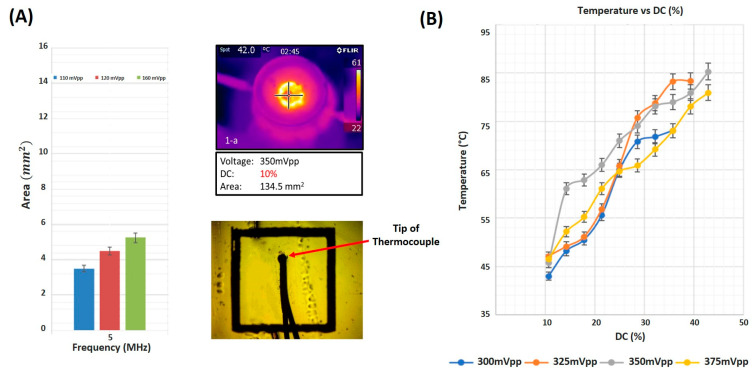
Focal area and temperature measurements. (**A**) Focal area as a function of amplitude of input signal. Heat distribution at the focus as captured by the IR camera and a close-up photo of the thermocouple in position. (**B**) Temperature measured by the thermocouple as a function of duty cycle at distinct input voltage amplitudes.

The focal area and temperature at the horizontal mid-plane varied as a function of increasing input voltage. When using a 5 MHz focused ultrasound transducer, the focal area increased from $3.5\;{mm}^{2}$ to $5.2\;{mm}^{2}$ as the input voltage increased from 110 mV to 160 mV (Figure 4A). Temperature measurements were acquired in real-time using a thread-head K-type thermocouple set at the focal area of the ultrasound transducer while sonicating at different voltages and duty cycles. While varying the input voltage, the temperature at the focal area increased as well with an increase in the duty cycle of ultrasound waves (Figure 4B). The increase was monotonic, recording a minimum temperature of 43 °C at the lowest input voltage of 300 mV and a DC of 10%, reaching the maximum temperature of 81.9 °C, when sonicating at an input voltage of 350 mV at the highest DC of 45%. The IR camera images emphasized a uniform distribution of thermal energy throughout the focal region of a recorded $134.5\;{mm}^{2}$ area when sonicating with ultrasound waves of 10% DC and an input voltage of 350 mV.

### 3.2. Cellular Ablation Using HIFU

#### 3.2.1. On 2D Cultures

**Figure 5:**
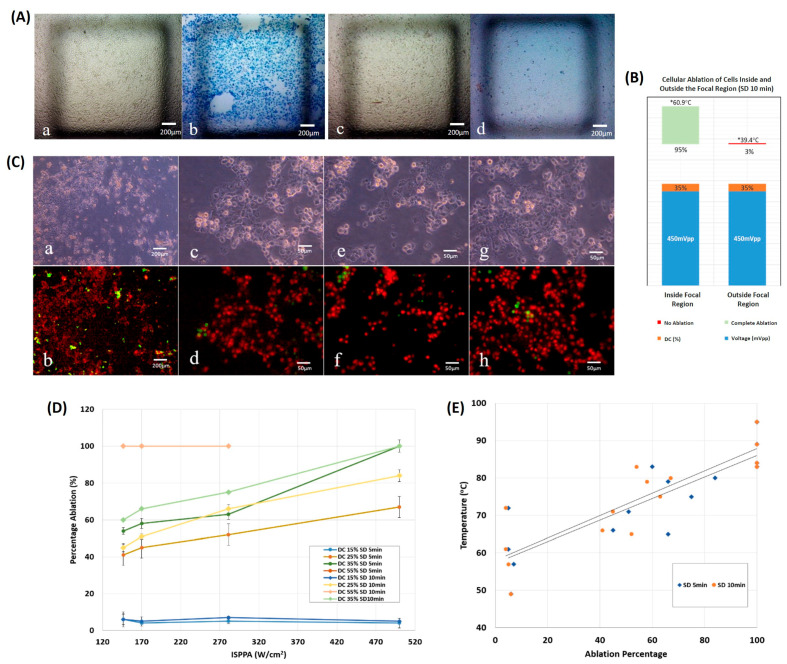
Monolayer ultrasonic ablation. (**A**) Bright field images, (**a**,**b**) cells located inside the focal region, pre-sonication and post-sonication, respectively, with dead cells stained in blue. (**c**,**d**) cells located outside the focal region, pre-sonication and post-sonication, respectively. (**B**) Ablation percentage following 10 min of sonication. The ablation area is greater than 95% inside the focal region, whereas it is in the vicinity of zero outside the focal region. (**C**) Bright field images of cells post sonication at (**a**) 5× magnification and at 20× magnification (**c**,**e**,**g**). Fluorescent images of cells at (**b**) 5× magnification and at 20× magnification (**d**–**h**). Results show 99% ablation where alive cells are stained in green, while dead cells are stained in red. (**D**) Percentage ablation of monolayers as a function of acoustic intensity at distinct DC and SD. (DC = 15, 25, 35 or 55%, SD = 5 or 10 min. (**E**) Final temperature versus ablation percentage of monolayers following ultrasound sonication for 5 or 10 min.

Ultrasound sonication was targeted toward a 2D layer of cells seeded on a glass coverslip, taking the configuration of a monolayer of cultured cancer cells. HIFU with a frequency of 2 MHz at a 35% duty cycle and $280\;W/{cm}^{2}$ intensity caused a 95% ablation of the 2D cultured cells inside the focal region, as opposed to a negligible 3% ablation outside the focal region. Bright field images of the cultured cells before and after ultrasound sonication for 10 min emphasized the difference in the ablation percentage (Figure 5A), with a maximum temperature of 60.9 °C, which was reached inside the area of focus, approximately 1.5 times higher than that achieved outside the transducer’s focus (Figure 5B).

Sonication duration and duty cycle of ultrasound waves affected the ablation percentage as a function of the applied spatial peak pulse average ultrasonic intensity (Figure 5D). Regardless of the sonication duration, a 15% low-duty cycle and a 55% high-duty cycle caused overlapping in the ablation percentage, which constantly varied as $I_{SPPA}$ increased. However, all cells were ablated at 55% DC, yet approximately less than 5% were ablated at 15% DC. In general, incrementing the sonication duration did not necessarily cause a significant change in ablation, contrary to increasing the duty cycle and $I_{SPPA}$. At 35% DC and at the highest $I_{SPPA}$ of $500\;W/{cm}^{2}$, full ablation of the cultured tumor cells occurred (Figure 5C). With the exception of the highest DC of 55%, a low $I_{SPPA}$ (≤170 $W/{cm}^{2}$) could only achieve an ablation percentage of less than 70% with all applied DC and SD. 

Majorly, the thermal ablation of cancerous cells cultured in 2D started at temperatures around 60 °C and reached full ablation at temperatures above 80 °C, regardless of the sonication time applied (Figure 5E). The ultrasound treatment time had a negligible effect on the temperature and ablation levels reached (p=0.557). The percentage of ablated 2D cultured cells increased monotonically with an increase in temperature.

#### 3.2.2. On 3D Cultures

**Figure 6:**
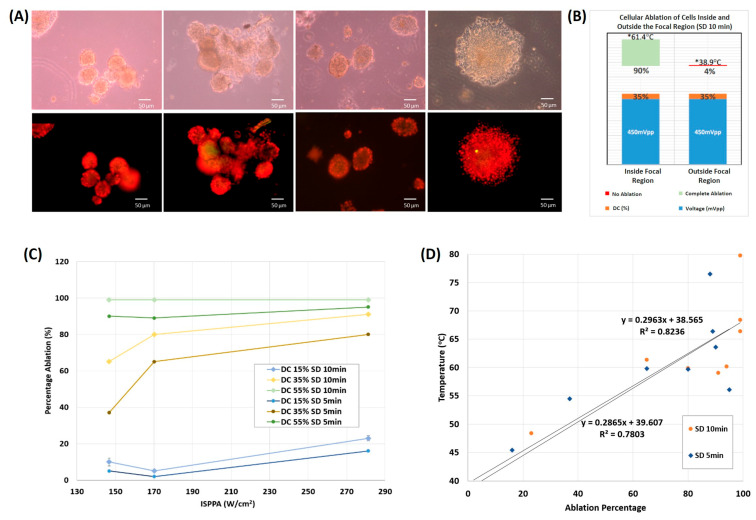
Spheroid ultrasonic ablation. (**A**) Bright field images of cells post sonication at 20× magnification (upper row). Fluorescent images of cells post sonication at 20× magnification (lower row). (**B**) Ablation percentage following 10 min of sonication. The ablation area is greater than 90% inside the focal region, whereas it is in the vicinity of zero outside the focal region. (**C**) Percentage ablation of spheroids as a function of acoustic intensity at distinct DC and SD (DC = 15, 25, 35 or 55%, SD = 5 or 10 min. (**D**) Final temperature versus ablation percentage of spheroids following ultrasound sonication for 5 or 10 min.

Cancerous cells 3D cultured in Matrigel underwent ultrasound sonication at a frequency of 2 MHz, with varying ultrasound intensity, duty cycle, and sonication duration. Bright field images and fluorescent staining of cells after sonication for 10 min with ultrasound waves of 35% DC, at an intensity of 280 $W/{cm}^{2}$, showed a significant 90% ablation percentage of tumor cells inside the focal region with an elevation in temperature to 61.4 °C, in comparison to a minimal 4% ablation percentage of cells recorded outside the field of focus with an approximate 1.57 times lower temperature reached (Figure 6B). Bright field images and fluorescent images at 20× magnification showed clusters of dead cells post-sonication, reaching up to 99% ablation at a high DC of 55% (Figure 6A).

The time of the ultrasound treatment affected the ablation percentage as a function of DC and $I_{SPPA}$ as well. At all parameters used, an SD of 10 min caused higher ablation percentages than an SD of 5 min. The full ablation of cancerous cells 3D cultured in Matrigel occurred at the highest DC used of 55%, after ultrasound sonication for 10 min, regardless of the applied ultrasound intensity (Figure 6C). A low 15% DC caused very minimal ablation (less than 20%) even after increasing SD and $I_{SPPA}$. In general, upon incrementing DC, SD, and $I_{SPPA}$, the percentage of ablated tumor cells increased, with a minimal dependence on SD at low and high DCs, in contrast with DCs in the middle range (35%) where spheroids reached higher ablation percentages as a function of both SD and $I_{SPPA}$.

At the end of each ultrasound sonication, 65% of the trials ablated cancerous spheroids at a temperature ≥60 °C (Figure 6D), reaching even higher ablation percentages and final temperatures with an increase in sonication time. Minimal ablation of spheroids was recorded at temperatures starting around 45 °C, following a monotonically increasing trend as the temperature increased.

### 3.3. Spheroid vs. Monolayer Thermal Culture Ablation

With the monolayer and spheroidal configurations, increasing DC significantly raised the percentage of ablated cells. A DC of 15% was the least efficient, whereas a DC of 55% caused the ablation of more than 90% of the cells, even at the lowest intensity. This was the outcome for both the monolayers (55% DC versus 15% DC: *p* = 0.008 for both SD = 5 min and 10 min) and spheroids (55% DC versus 15% DC: *p* = 0.022 and 0.028 for SD = 5 and 10 min, respectively). However, as long as SD was considered, and in both configurations, similar ablation percentages were achieved with no significant difference at a given DC and $I_{SPPA}$. The Mann–Whitney U-test yielded insignificant results when comparing ablation percentages at an SD of 5 min to an SD of 10 min, at a DC of 15% (monolayers: *p* = 0.2; spheroid: *p* = 0.4), and at a DC of 55% (monolayers: *p* = 1.0; spheroids: *p* = 0.2).

**Figure 7:**
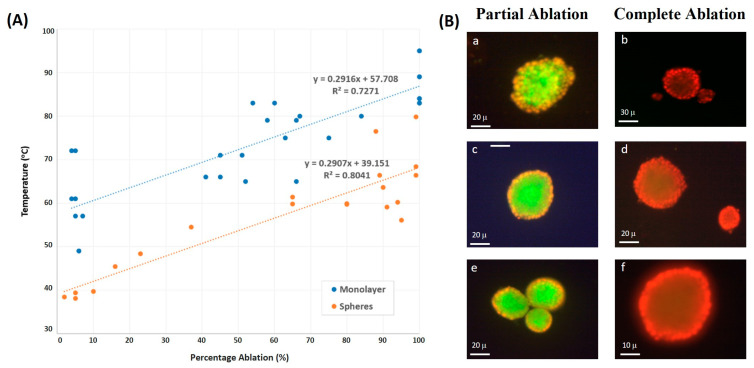
Two-dimensional vs. three-dimensional culture ablation. (**A**) Final temperature versus ablation percentage of monolayers and spheroids for distinct combinations of DC and SD. (**B**) Microscope images of different spheroids post sonication. With partial ablation (**a**,**c**,**e**), the outmost surface showed red stains (dead cells), whereas the cells closer to the center remained alive. With complete ablation (**b**,**d**,**f**), the red color dominates.

The ultrasound sonication of tumor cells with a 2D monolayer configuration determined their ablation at temperatures higher by 20 °C than those cultured in Matrigel, taking a 3D spheroid configuration (Figure 7A). Spheroids needed lower temperatures than monolayers to reach the same ablation percentage at all applied ultrasound intensities. Add to that, the correlation between the temperature and percentage of tumor ablation showed less scattering with spheroids in comparison to monolayers of cells, emphasizing that spheroids are less sensitive to changes in temperature.

Images using Fluorescent Cyto3D Live/Dead Assay of spheroids post ultrasound sonication showed partial ablation of the cultured cells, which is determined by the death of the outermost layer (stained in red), while the innermost layer remained intact (stained in green) when sonicating at lower DC values. However, at the end of ultrasound sonication with higher DC and $I_{SPPA}$, the complete ablation of spheroids was achieved (Figure 7B), where most cells in each cluster, whether in the outermost layer or the innermost one, were fully ablated after absorbing more power per unit area.

### 3.4. Numerical Simulations

In this section, the obtained experimental and numerical results are presented for the different operating conditions in terms of DC and transducer pressure levels to analyze their effect on the ablation rate for the different cell culture types (monolayer and spheroids).

The developed mathematical models were first validated against the experimental data for both cell cultures. To that end, the model was simulated using the geometry of the adopted transducer in the experiment with a focal distance of 51.4 mm, an external diameter of 33 mm, and a fundamental frequency of 2 MHz. Our computational model describing the ablation of 2D and 3D cultured cells showed agreement with the experimental results in terms of the maximum temperature reached at the focal region within the cell cultures with a maximum error of 15%, as shown in Table 2. Relatively large errors were obtained between the numerical and experimental data, with the numerical values having higher temperature levels. This overestimation of the thermal response and the large error could be attributed to neglecting the cavitation effect due to water vapor evaporation, which requires significant heat and could reduce cell temperature. In addition, mechanical deformation of the cells could alter the culture domain and cause variation in thermal energy diffusion. Nonetheless, an error of 15% is deemed acceptable in practice, and the model was considered reliable for further analysis.

**Figure 8:**
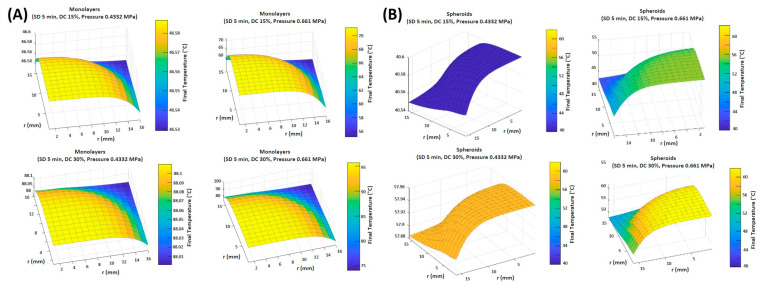
Range of final temperatures (°C) in the plane passing through the cells modeled as (**A**) monolayers and (**B**) spheroids at a DC of 15% and 30%, and a pressure of 0.433 MPa and 0.661 MPa after the simulation of ultrasound sonication for 5 min.

In both the monolayer and spheroid simulations, surface plots spanning one-quarter of the disk demonstrated a uniform distribution of thermal energy at low-pressure runs of 0.433 MPa, with a minimal temperature gradient (less than 0.1 °C) between the focal region and the remainder of the culture medium. This was achieved with both low and high DC values (Figure 8A,B). However, at high-pressure runs of 0.661 MPa, a large temperature gradient was recorded between the focal region and its immediate surroundings, reaching up to 21.7 °C, computed at the 30% high DC in the monolayer configuration, and up to 14 °C difference computed at a DC of 30% with the spheroid configuration (Table 3). Nevertheless, within the focal region, the temperature distribution was relatively uniform, with a negligible temperature gradient.

The configuration of the cultured cells did not affect how the latter interacted with the thermal energy induced by ultrasound sonication. Both monolayer and spheroid cell cultures showed similar behaviors regarding thermal responses to the sonication conditions. However, higher temperature values were attained when using the monolayer culture compared to the spheroids for the same sonication parameters. As was demonstrated in the experimental work, a significant difference of up to 20 °C was recorded between the temperature reached by monolayers and that by spheroids. This difference in temperature was more apparent as DC increased.

## 4. Discussion

High-intensity focused ultrasound has been widely used as a novel technology for cancer treatment due to its minimal invasiveness, cost-effectiveness, and high precision. Our research focused on the effect of pulsed HIFU on 2D and 3D breast cancer cell culture models to explore the effect of cell configuration and ultrasound parameters on the efficiency of HIFU for ablating breast cancer cells. A HIFU treatment was considered efficient if it led to an ablation percentage greater than or equal to 90%. Our investigation delved comprehensively into the thermal ablation of 2D and 3D cancer cell cultures across a spectrum of ultrasound intensities, duty cycles, and sonication durations. Key metrics of interest included the percentage of cellular ablation and the maximum temperature reached during ablation. By investigating the response of these two culture models to ultrasound sonication, we aimed to characterize the optimal ultrasound parameters necessary for effective cancer cell ablation, which can help clinicians optimize treatment protocols and ultimately improve patient outcomes.

Furthermore, we developed a mathematical model to elucidate how cells in monolayer and spheroid cultures respond to pulsed HIFU. These simulations were subsequently compared with the experimental data to validate the model’s accuracy. This integrated approach helped predict cellular ablation outcomes under varying conditions, encompassing alterations in duty cycles and maximum pressure levels, which would eventually facilitate the translation of HIFU treatments from preclinical studies to clinical trials with greater confidence in their effectiveness and safety.

### 4.1. Duty Cycle Affects Degree of Cellular Ablation

The duty cycle in ultrasound sonication denotes the fraction of the burst period during which ultrasound amplitude is nonzero, referred to as the ON region. A higher DC corresponds to an extended duration in which cells can absorb energy, resulting in a higher energy input per unit area. Conversely, a lower DC corresponds to a shorter ON region and a more extended OFF region, during which the absorbed energy dissipates into the surrounding control volume encompassing the target.

In both monolayer and spheroidal configurations, the percentage of ablated tumor cells was highly influenced by the DC of ultrasound waves, which increased as a function of increasing DC. When targeting bovine liver tissue in vitro with a 40 kHz frequency difference between the inner and outer loops of a dual-frequency transducer at 160 W ultrasonic power, Zhu et al. [56] reported a significant change in the structural form and dimensions of the lesion as DC was changed. With low DC values, ranging between 5 and 20%, no coagulation necrosis was recorded, unlike with a DC of 30% and above, where coagulation necrosis appeared, with the highest ablation percentage occurring at a DC of 50%. Similarly, our study showed that a low DC of 15% was totally inefficient, while a DC of 55% caused the ablation of more than 90% of the tumor cells. Increasing DC elongated the heating time and diminished the dissipation time, which is characterized by a shorter inactive treatment interval, allowing thermal ablation to dominate as more shock wave arrays continued to hit the targeted tissue [57]. Indeed, higher temperatures were attained with higher DC values as a result of longer heat depositing intervals. However, there is a potential trade-off. Increased ultrasonic pulse frequency, driven by higher DC values, elevates the risk of inducing mechanical disintegration and localized fragmentation of the targeted tissue due to inertial cavitation, as noted in prior research [58]. After a certain threshold, even with further increases in DC values beyond those employed in our study, a transition occurs wherein the efficacy of thermal ablation begins to diminish, leading to the predominance of mechanical ablation as the more efficient process.

Our findings revealed that applying ultrasound sonication for durations of 5 min or 10 min yielded nearly similar ablation percentages, with negligible differences, particularly at low and high DC values for both monolayers and spheroids. This observation led to the hypothesis that cells might reach a state of equilibrium in terms of power absorption and energy dissipation before the conclusion of the sonication session, indicating a saturation in behavior at an optimal exposure duration. Consequently, a 5 min treatment duration may be considered more efficient, as it achieves the desired ablation percentages with less time and energy expenditure.

### 4.2. Monolayer vs. Spheroid Ablation Temperature

Cells modeled as spheroids were ablated at a threshold temperature significantly lower than that of monolayers (approximately 20 °C less), demonstrating the decreased sensitivity of spheroids to thermal ablation. In general, spheres have a higher surface area and more pathways for heat dissipation compared to monolayers, where heat is more evenly distributed across the flat surface. The additional dimension in spheres could contribute to enhanced heat dissipation. They also have a lower surface-to-volume ratio compared to flat monolayers. This can affect the efficiency of heat dissipation, as a higher surface-to-volume ratio allows for more efficient cooling. Both 2D and 3D cell cultures serve as effective in vitro models, and the observed differences in ablation temperatures indicate that for the same ablation percentage, higher temperatures are needed to ablate 2D cell cultures [59]. Both cell configurations differed in oxygen and nutrient availability and distribution among the cells in each cluster. During treatment, the cells absorbed the acoustic intensity in the ON region for further energy dissipation to the surroundings during the OFF region. In general, spheroids have a cell growth medium with a higher thermal conductivity than that of monolayers. They also have a higher surface-to-volume ratio. Thus, cells in the innermost core of the spheroids would dissipate power into the surrounding cells during the OFF region, unlike cells in monolayers where the dissipated power would mostly spread across to the surrounding cell growth medium.

The results showed a uniform distribution of heat across the focal region in both cell configurations. However, approximately 12.5% higher maximum intensity was recorded at the same applied pressure in spheroids than in monolayers, which increased as the applied pressure was also increased [60]. This can potentially be due to an additional absorption coefficient and specific heat capacity offered by the hydrogel to spheroids (Table 1), causing the latter to absorb much more heat than the hydrogel-lacking monolayers while only leading to a slow increase in temperature. This could explain why higher intensities causing higher temperatures were required to implement the same ablation percentage in the monolayer case. For the same sonication parameters, however, the spheroids significantly heated up at lower temperatures, implying they were more susceptible to mechanical ablation than thermal ablation at this stage compared to monolayers due to microenvironment disruption altering tumor cell viability [61]. With high negative pressures and shorter HIFU pulses [62], residual gas bubbles present within the spheroids produced cavitating bubbles that caused mechanical damage rather than thermal damage in a series of cavitation events [63].

### 4.3. Partial and Complete Cellular Ablation in Spheroids

The cells at the outermost layer of the spheroids were ablated before those in the innermost layer, which remained intact at low DC and sonication intensities. As DC and intensity were increased, full ablation of all the cells in the clusters was achieved at the end of each sonication session.

Spheroids have an oxygen concentration gradient from the outer surface to the inner core, with cells located on the outermost surface of the cluster being exposed to oxygen more than those at the core, which became hypoxic spots [18]. Hence, the latter were more susceptible to ablation at higher intensities while reaching higher temperature levels. Nonetheless, microscopic images showed that the outer surface of a cell cluster ablated first, followed by the hypoxic core, suggesting a gradient in the distribution of ultrasound power per unit volume while being transferred across the cells. In general, 3D cell cultures have a more intricate interconnection between cells, resulting in the formation of gradients in pH, oxygen concentration, and metabolic activity [64]. These cell–cell interactions could form a shielding effect on the cells present at the core despite the uniform thermal distribution across.

### 4.4. Model Validation

Enhancing the efficiency of HIFU treatment involves the simulation of non-linear acoustic wave propagation through diverse layers of media with an ultrasound transducer. These simulations involve experimentation with various techniques to precisely focus the waves, gauging their impact on increasing the temperature within the targeted tissue and, consequently, inducing lesion formation. These computational studies play a crucial role in planning and optimizing HIFU procedures intended for clinical application.

Our computational model highlighted the difference in the maximal temperature reached by both simulated cell configurations. Specifically, spheroids were subjected to a higher heat deposition due to their higher acoustic impedance (higher density of spheroids culture media (RPMI) compared to that of the monolayer (DMEM)). Moreover, spheroids, in general, are characterized by a thicker layer (117 μm vs. 15 μm), which increases their total impedance. For these reasons, higher acoustic energy was deposited in the cells, resulting in higher heat fixation. Nonetheless, the temperature of the spheroids was significantly lower even though both culture media had similar specific heat capacities. This could be attributed to the 75% higher conductivity of spheroids gel (0.53 W/K·m) compared to that of the monolayer (0.13 W/K·m). This resulted in higher heat conduction and dissipation to the surroundings, leading to reduced temperature levels recorded with the spheroids. This effect, combined with the additional specific heat capacity from the hydrogel medium, contributes to a slower increase in temperature within the spheroids compared to the monolayers. The temperatures were reported in the plane of the cells’ medium at the end of the sonication period. Temporal variations in the temperature for both 2D and 3D cultures are expected to be similar due to the similar thermal characteristics of the cultures, mainly the specific heat capacity. The latter is the main factor affecting the heat storage of the cells, and consequently, their temperature rises. For similar $C_{b}$, the cultures are expected to have similar temperature variation with time, and thus, the same thermal dose. Accordingly, the thermal dose value will be affected by the actual temperature levels instead of the temporal gradient.

Furthermore, at low DC, the spheroids were ablated at the boundary first while the inner cells remained intact. Upon the increase in DC, all the cells were ablated, including the innermost ones. Our model showed that the temperature of the spheroids was uniform throughout the culture medium with no significant temperature gradient within the focal region. Similar observations were obtained by Zhou et al. [65], where the center of the ablated cancer appeared analogous to viable cells after H&E staining. These cells retained their cytologic staining and nuclear chromatin features with no evidence of deterioration. Nevertheless, electron microscopy revealed vacuoles in the cytoplasm of apparently normal cancer cells after HIFU treatment. These vacuoles contained disintegrated cell membranes and unidentified organelle structures. This implies an irreversible cell death due to thermal fixation rather than incomplete coagulation necrosis [65]. The central region of the ablated tumor remained intact due to limited wound healing in the immediate aftermath of HIFU treatment. Conversely, the peripheral region displayed typical signs of lethal and irreversible cell damage, resembling coagulation necrosis. These findings highlight the superiority of NADH-diaphorase staining over H&E staining for assessing acute cell death. While H&E relies on changes in cellular structure, NADH-diaphorase focuses on the presence or absence of enzyme function, providing a more precise and unbiased assessment. Consistent with these observations, Wang et al. reported lethal damage in the peripheral region and a normal appearance with preserved cell structure in the central region, suggesting thermal fixation [66].

However, to explain such a phenomenon, assuming the staining observations were reliable, one possible reason that could be attributed to such findings is the effect of mechanical ablation instead of thermal ablation at this level with the application of pulsed HIFU. Mechanical effects could result in the ablation of spheroids at lower temperatures compared to monolayers. In fact, using high-intensity ultrasound waves, mechanical effects, such as cavitation and microstreaming, are dominant [67]. Pulsed HIFU can induce bubble formation that disrupts the tissues, leading to liquefaction after the bubble explodes due to subsequent acoustic waves. Such mechanical effects are more prominent when pulsed HIFU beams are used instead of continuous HIFU, where the burst duration or pulse length is shorter than the time needed for the cells to boil, form a bubble, and then burst [68]. Accordingly, bubbles that form on the inner or central region could be protected by the cell–cell and cell–matrix interactions, thus resisting HIFU ablation more than the outermost cells where the bubbles could be easily depleted.

The formation of microbubbles in spheroids could also explain the difference in temperature levels attained in this 3D cell culture compared to the monolayer. Additionally, the bubbles formed by HIFU could potentially create a shielding layer that prevents the HIFU wave penetration into the core, causing the waves’ reflection and backscatter, thereby reducing the amount of thermal energy dissipated in the cells [69,70]. Such acoustic wave reflection could not be captured by the adopted acoustic wave propagation model since it neglects the formation of the bubbles. Moreover, the formation of these bubbles may be limited in the monolayer culture due to its 2D geometry and small thickness compared to the more realistic 3D spheroid culture.

### 4.5. Limitations

Our study had some limitations whereby all interactions of cells with the extracellular medium, similar to an in vivo setting, were not taken into consideration. Cell deformation due to mechanical shocks and cavitation may take place using HIFU ablation, especially in pulsed mode. However, they were not considered in this work, which could lead to overestimation of the actual temperature levels. In addition, temperature measurements were highly dependent on the sensitivity of the thermocouple used and its right positioning in the focal region without damaging the cultured medium. Note that the same Petri dish contained varying sizes of spheres, posing challenges when examining how the HIFU parameters affected spheroid dimensions. We were also unable to quantify the number of cells before subjecting them to sonication. Nonetheless, our work emphasized the ability of HIFU to ablate 2D and 3D cultured tumors while identifying the effect of ultrasound parameters on the ablation percentage, area of damage and temperature rise post sonication.

## 5. Conclusions

Our study focused on the experimental and computational analysis of HIFU thermal ablation in breast cancer cells, examining two cellular configurations: 2D monolayers and 3D spheroids, both considered effective in vitro models. The study investigated the effect of HIFU parameters such as acoustic intensity, duty cycle, and sonication duration on effective tumor ablation while minimizing damage to surrounding healthy tissues. Our findings highlighted the distinct responses of 2D monolayers and 3D spheroids to HIFU treatment, with spheroids exhibiting a lower temperature threshold for effective ablation and a significant increase in ablation percentage with elevated duty cycles. The integration of experimental and computational approaches yielded a strong understanding of the variation in HIFU ablation results across the two models. Additionally, the study’s focus on 3D spheroid models emphasized the significance of capturing complex cellular interactions. This underscores the need for advanced model systems that more accurately mimic the tumor microenvironment to enhance preclinical assessments.

## Figures and Tables

**Table 1:** Thermal and acoustic properties of the different layers of cell cultures.

Layer	ρ (kg/m^3^)	C (J/kg∙K)	k (W/m∙K)	$c_{0}$ (m/s)	$\alpha_{ref}$ (Np/m)	B/A (−)	η (−)
Water	1000	4180	0.615	1520	2.8 × 10^−4^	3.5	2
DMEM	1023	3800	0.13	1560	-	-	-
Hydrogel	1060	3770	0.53	1560	-	-	-
Cells	1066	3610	0.442	1423	0.57	-	0.75
Glass	2500	880	1.1	4500	0.27	6.8	1.32
Petri Dish	1000	1350	0.165	2000	0.27	6.8	1.32

**Table 2:** Comparison between the experimental and numerical results.

Configuration	DC (%)	Pressure at the Source (MPa)	Experimental Maximum Temperature (°C)	Numerical Maximum Temperature (°C)	Error (%)
Monolayer	15	0.433	49.0	46.6	5.0
		0.661	72.0	71.2	1.1
	30	0.433	74.5	88.1	15.0
Spheroids	15	0.433	38.0	40.6	6.8
	30	0.433	54.5	58.0	6.4
	55	0.433	63.3	63.5	0.3

**Table 3:** Final temperature range.

Configuration	DC (%)	Pressure at the Source (MPa)	Final Temperature Range (°C)
Monolayer	15	0.433	(46.53; 46.59)
		0.661	(55.13; 71.21)
	30	0.433	(88.00; 88.11)
		0.661	(74.01; 95.71)
Spheroids	15	0.433	(40.55; 40.6)
		0.661	(42.9; 55.41)
	30	0.433	(57.88; 57.96)
		0.661	(48.01; 62.01)

## Data Availability

Data are contained within the article.
